# Hospital Meetings

**Published:** 1903-02-21

**Authors:** 


### HOSPITAL MEETINGS.

### ST. MARK'S HOSPITAL.

The annual general meeting of St. Mark's Hospital for Fistula and other diseases of the rectum was held at the Mansion House on Thursday morning (12th inst.). The business was purely formal. Mr. R. B. Martin (treasurer) was in the chair, supported by Mr. E. F. Carey and Mr. Hill.

The adoption of the report and accounts was moved from the chair. The small attendance was taken by the chairman to be a sign of confidence in the committee. He would, however, have been glad to see more subscribers and donors, as an evidence of their interest in the affairs of the hospital. He was very glad that the Lord Mayor had followed the example of his predecessors in becoming president during his year of office; the hospital had always been connected with the City, and it was to be hoped this would continue. The festival dinner, presided over by the Lord Mayor, who was supported by the Sheriffs, produced £1,621 15s. 9d. Although he would have liked more it was sufficient to leave a good sum in hand. He would remind subscribers that though the hospital was, he thought, in an extremely good and satisfactory hygienic condition, and eminently capable of treating the diseases for which it was originally designed, and though all available skill and necessary appliances were provided, yet more money was required if the beds were to be filled and the cost per head reduced. He was not one of those who would diminish the cost per bed at the expense of the patients' comfort. It was a pity that the beds were not filled, when the hospital possessed such eminently suitable appliances. The hospital suffered from its position: it was in a very useful place in some respects, but out of the way as far as people of more leisure than City men were concerned. Situated in the West-end of London it would have more attention.

Sir Alfred Cooper seconded and the motion was carried.

A list of votes of thanks to the various honorary officers connected with the hospital followed, including one to the Ladies' Committee of the Samaritan Fund, proposed by Mr. A. Leviter, and seconded by Sir H. Crawford. Mr. J. Edmonds, after visiting many Paris hospitals, expressed the opinion that St. Mark's was "a perfect hospital." Nothing, he said, could exceed the kindness and skill of the surgeons, and he did not believe there was a farthing's waste.

### ROYAL LONDON OPHTHALMIC HOSPITAL.

The Lord Mayor and the Lady Mayoress visited the Royal London Ophthalmic Hospital, City Road, on the 16th inst., and formally opened 30 additional beds, towards the maintenance of which a grant has been made by King Edward's Hospital Fund. Among those present were Miss Samuel, Alderman and Sheriff Sir George Truscott, Sheriff Sir Thomas Brooke-Hitching, Mr. H. Davison (vice-chairman of the committee of management), and Mr. R. J. Bland (secretary).

The Lord Mayor, in declaring the beds open, referred to the immense amount of good which had been done by King Edward's Hospital Fund. He said that it set a seal upon those institutions which could show good management, and it withheld subscriptions from those institutions which were improperly managed. The Fund had assisted the Royal London Ophthalmic Hospital by a further annual grant of £1,100 and a special donation of £2,500 for the opening of 30 more beds. The Chief Rabbi, (Dr. Adler) made a short speech, and Mr. Davison then moved a vote of thanks to the Lord Mayor and the Lady Mayoress. He announced that the Lord Mayor had made a donation to the hospital of 20 guineas, and each of the Sheriffs had given 10 guineas. They had also received a donation of £100 towards the maintenance of two beds through the chairman of the Royal London Ophthalmic Hospital Guild. Although they had altogether 138 beds, only 70 could be opened when they first started work in the present buildings. When appealing for funds they were often met with the reproach that they should not have left the old Moorfields site, where they had lived almost rent free and were subject to a nominal amount for rates, in order to move to a new site where, with a larger building they incurred much heavier expenses. The committee were prepared for increased expenditure, but they did not expect that the rating authorities would treat the hospital on a different basis from that of similar institutions in the same district. As to the question of removing the hospital to the present site, he thought that no one could say that the committee had not acted in a business-like way. The last bill had been paid, the Building Committee no longer existed, and they had closed the account with a credit balance of nearly £600. The Lord Mayor, in replying to the vote of thanks, said that the policy of removing hospitals was one which had of late come into great prominence. In the case of this institution they had received £78,500 for the old site, and if they had not sold it they must have rebuilt the hospital on the old site, with the result that they would not, perhaps, have been able to add a single bed. In his opinion it was not a question as to whether a lot of harm had been done, but whether a great deal of good had resulted from the movement. With regard to the rating of hospitals, he said that the time was comin[illegible] when the question would have to be considered whether t[illegible] authorities would not exempt the hospitals instead of gett[illegible] all that they could from them.

